# Abnormalities of signal transduction networks in chronic schizophrenia

**DOI:** 10.1038/s41537-017-0032-6

**Published:** 2017-09-12

**Authors:** Jennifer L. McGuire, Erica A. Depasquale, Adam J. Funk, Sinead M. O’Donnovan, Kathryn Hasselfeld, Shruti Marwaha, John H. Hammond, Vahram Hartounian, James H. Meador-Woodruff, Jarek Meller, Robert E. McCullumsmith

**Affiliations:** 1 Department of Psychiatry and Behavioral Neuroscience, University of Cincinnati, Cincinnati, OH USA; 2 Department of Biomedical Informatics, Cincinnati Children’s Hospital Medical Center, Cincinnati, OH USA; 3 Department of Molecular and Cellular Physiology, University of Cincinnati, Cincinnati, OH USA; 4 Department of Psychiatry and Behavioral Neurobiology, University of Alabama-Birmingham, Birmingham, AL USA; 5 Psychiatry & Neuroscience, The Icahn School of Medicine at Mount Sinai, New York, NY USA; 6 James J. Peters VA Medical Center, Mental Illness Research Education and Clinical Center (MIRECC), Bronx, NY USA; 7 Departments of Environmental Health, Electrical Engineering & Computing Systems and Biomedical Informatics, University of Cincinnati College of Medicine, Cincinnati, OH USA

## Abstract

Schizophrenia is a serious neuropsychiatric disorder characterized by disruptions of brain cell metabolism, microstructure, and neurotransmission. All of these processes require coordination of multiple kinase-mediated signaling events. We hypothesize that imbalances in kinase activity propagate through an interconnected network of intracellular signaling with potential to simultaneously contribute to many or all of the observed deficits in schizophrenia. We established a workflow distinguishing schizophrenia-altered kinases in anterior cingulate cortex using a previously published kinome array data set. We compared schizophrenia-altered kinases to haloperidol-altered kinases, and identified systems, functions, and regulators predicted using pathway analyses. We used kinase inhibitors with the kinome array to test hypotheses about imbalance in signaling and conducted preliminary studies of kinase proteins, phosphoproteins, and activity for kinases of interest. We investigated schizophrenia-associated single nucleotide polymorphisms in one of these kinases, AKT, for genotype-dependent changes in AKT protein or activity. Kinome analyses identified new kinases as well as some previously implicated in schizophrenia. These results were not explained by chronic antipsychotic treatment. Kinases identified in our analyses aligned with cytoskeletal arrangement and molecular trafficking. Of the kinases we investigated further, AKT and (unexpectedly) JNK, showed the most dysregulation in the anterior cingulate cortex of schizophrenia subjects. Changes in kinase activity did not correspond to protein or phosphoprotein levels. We also show that AKT single nucleotide polymorphism rs1130214, previously associated with schizophrenia, influenced enzyme activity but not protein or phosphoprotein levels. Our data indicate subtle changes in kinase activity and regulation across an interlinked kinase network, suggesting signaling imbalances underlie the core symptoms of schizophrenia.

## Disease mechanisms: A signaling imbalance

A study by US scientists indicates that changes in the activity of key signaling proteins may underlie core symptoms of schizophrenia. Protein kinases mediate the activation of intracellular signaling events and analyses of the kinome, the complete set of protein kinases encoded in the genome, previously revealed significant changes in phosphorylation patterns in postmortem brain tissue from patients with schizophrenia. Based on these findings, Jennifer McGuire at the University of Cincinnati and colleagues investigated the upstream regulation of these proteins. They identified both established and novel proteins associated with schizophrenia in the anterior cingulate cortex, with JNK and AKT activity being the most disrupted in schizophrenia patients. Their findings highlight how subtle changes in the activity of a small number of signaling proteins can propagate and have major consequences for mental health.

## Introduction

Schizophrenia is a serious cognitive disorder of unknown etiology. Gene expression, cytoskeletal organization, neurotransmitter systems, and more, are implicated in schizophrenia pathophysiology.^1, 2^ These processes are governed to varying extents by kinase-mediated signaling events. Intracellular signaling is traditionally described as “pathways” or “cascades,” implying a linear sequence of molecular events. However, the identification of signal integration molecules and insights into “crosstalk” between signaling molecules indicate these “pathways” are, more accurately, complex and dynamic networks.^3^ Signaling networks often converge on multi-potent signaling molecules, such as DARPP-32, which integrate input from multiple neurotransmitter receptor subtypes. We postulate that schizophrenia may be a disorder mediated by subtle changes in signaling networks affecting multiple domains, including cell metabolism, molecular trafficking, inter-cellular signaling, and the functional integrity of neurocircuits.

Previously, we reported altered serine–threonine kinase activity in schizophrenia using a kinome array chip adapted for use with postmortem brain.^4^ Using this data set, we developed a novel bioinformatics protocol identifying kinases involved in the pathophysiology of schizophrenia. We performed additional array experiments testing the extent to which chronic antipsychotic medication alters kinase activity in brain homogenate from haloperidol decanoate-treated rats. We confirmed our results using biochemical assays and kinase inhibitors. Finally, we demonstrated that the schizophrenia-linked single nucleotide polymorphism (SNP) of protein kinase B (AKT), rs1130214, may affect AKT enzyme activity.

## Results

**Fig. 1:**
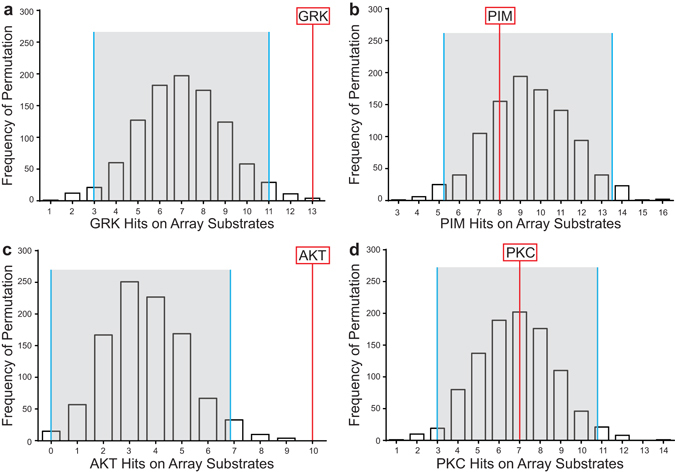
The observed frequency of selected kinases relative to the expected distribution in schizophrenia versus control subjects (**a**, **b**) and haloperidol versus vehicle-treated rats (**c**, **d**). The distribution is derived from 2000 randomly sampled same-size subsets from the kinome array. *Red lines* indicate the number of times the kinases is observed in the schizophrenia or haloperidol data set. *Gray* areas indicate +/−2 standard deviations from the expected distribution mean. Abbreviations: G-protein-coupled receptor kinase (GRK; proto-oncogene serine/threonine protein kinase (PIM); protein kinase B (AKT); protein kinase C (PKC)

Previously, we identified 19 peptide sequences with +/−1.15 fold-change difference in kinase activity between schizophrenia and control subjects (Supplementary Tables S1, S2).^4^ As we were the first group to use this hypothesis-generating platform to study postmortem brain, there is not a clear consensus in the field for what magnitude of fold-change is biologically relevant. We based our initial fold-change threshold (+/−1.15) on preclinical studies showing alterations in downstream biological functions in this range.^5–8^ For example, changes in kinase activity within this range potently alter protein synthesis.^5^ In the present study, we assigned upstream kinases to these 19 differentially phosphorylated peptides and generated frequency distributions for potential kinases using random sampling analysis (Fig. 1). Representative probability plots are shown for one of our significant hits (GRK, Fig. 1a) and one kinase that was not overrepresented (proto-oncogene serine/threonine protein kinase (PIM), Fig 1b) in schizophrenia. In contrast to GRK, PIM falls near the mean of the expected distribution (Fig. 1b). From this analysis of all 19 substrates, we identified 7 overrepresented kinases: p21-associated kinases (PAK), G-protein-associated kinases (GRK), protein kinase A (PKA), casein kinase (CK), protein kinase D (PKD), dystrophia myotonica protein kinases (DMPK) and never in mitosis gene A-related kinases (NEK) from the original set of 19 peptides altered in schizophrenia (Supplementary Table S3).

### Effects of haloperidol on kinase activity

We predicted that 9 months of haloperidol administration would affect serine–threonine kinase activity in rats. 16 substrates exhibited +/−1.15 or greater fold-change (Supplementary Table S2). Four substrates overlapped between the schizophrenia and haloperidol data sets. Interestingly, fold-change of all overlapping peptides was in opposite directions (Supplementary Table S3). We performed random sampling on the haloperidol rat data set using 2000 iterations of 16 randomly selected peptides which identified three overrepresented kinases, PKA, DMPK, and AKT altered by haloperidol treatment (Supplementary Table S3). Representative probability plots are shown for one of our significant hits (AKT, Fig. 1c) and one kinase that was not overrepresented (PKC, Fig. 1d) in schizophrenia. In contrast to AKT, PKC falls near the mean of the expected distribution (Fig. 1d).

### Pathway analysis

**Fig. 2:**
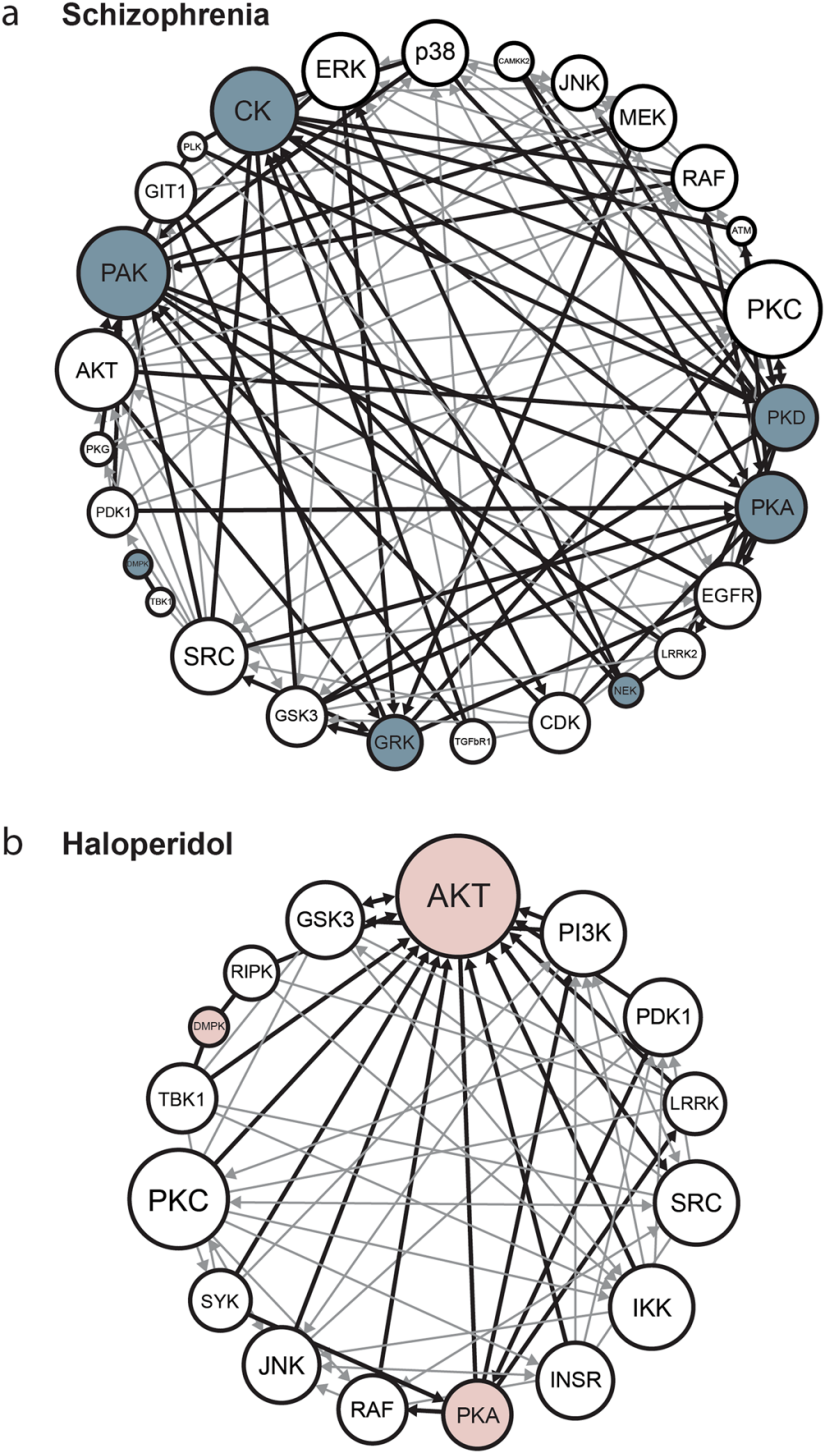
Kinases implicated by the random sampling analyses (*gray circles*) were used to create larger schizophrenia (**a**) or haloperidol (**b**) interaction networks. Using Ingenuity Pathway Analysis, we added kinases directly acting on our kinases of interest (*thicker lines*). Known direct interactions were established in Ingenuity between all members of the emerging network (*thicker and thinner line*s). Weakly connected kinases (two or fewer connections) were removed to create a core network of kinases. Abbreviations: p21-activated kinases (PAK); Protein kinase D (PKD); G-protein-coupled receptor kinase (GRK); dystrophia myotonica-protein kinase (DMPK); casein kinase (CK); protein kinase A (PKA); never in mitosis gene A-related kinase (NEK); protein kinase B (AKT); protein kinase C (PKC); c-Jun N-terminal kinase (JNK); protein kinase G (PKG); cyclin-dependent kinase (CDK); p38 mitogen-activated protein kinase (p38); calcium/calmodulin-dependent protein kinase kinase 2 (CAMKK2); mitogen-Activated protein kinase kinase (MEK); extracellular signal-regulated protein kinase (ERK); phosphoinositide-dependent kinase 1 (PDK1); GPCR kinase-interacting protein 1(GIT1); polo-like kinase (PLK); glycogen synthase kinase 3 (GSK3); proto-oncogene tyrosine-protein kinase SRC (SRC); TANK-binding kinase 1 (TBK1); transforming growth factor beta receptor 1 (TGFbR1); epidermal growth factor receptor (EGFR); leucine-rich repeat kinase (LRRK2); rapidly accelerated fibrosarcoma (RAF); serine protein kinase ATM (ATM); spleen tyrosine kinase (SYK); I kappa B kinase (IKK); insulin receptor (INSR); receptor-interacting protein kinase (RIPK); phosphoinositide 3-kinase (PI3K)

We investigated the larger signaling environment within which these kinases function using ingenuity pathway analyses (IPA, Qiagen) to identify linked kinases. IPA identified components of ERK and AKT signaling (RAF/MEK/ERK, and PDK1/AKT/GSK3, respectively) as directly interacting with schizophrenia kinases (Fig. 2a). Our haloperidol data set indicated interactions with components of AKT signaling, including PI3K, PDK1, IKK and GSK3 (Fig. 2b).

Not surprisingly, IPA identified phosphorylation post-translational modification as the top function of schizophrenia and haloperidol kinase networks (*p* = 1.99E-57 and *p* = 1.36E-38, respectively) (Supplementary Table S4). The schizophrenia network aligned with cytoplasmic organization (*p* = 1.1E-25), cytoskeleton (*p* = 2.45E-24) and microtubule dynamics (*p* = 2.47E-20).^4^ Schizophrenia kinases associated with neurite growth (*p* = 6.13E-17), neuronal differentiation (*p* = 3.7E-10), and long-term potentiation (*p* = 5.57E-09). The haloperidol network overlapped with mechanisms of cell death and survival (*p* = 9.06E-26-5.12E-06) and was functionally linked to B-lymphocyte viability (*p* = 3.48E-17), platelet aggregation (*p* = 9.25E-16) and immune cell proliferation (*p* = 4.55E-14).

Using IPA, we compared our networks against canonical signaling cascades. Schizophrenia kinases aligned with ErbB (*p* = 3.47E-43), gonadotropin-releasing hormone (*p* = 1.94E-38), and renin-angiotensin (*p* = 3.62E-38) signaling (Supplementary Table S5). The haloperidol kinases overlapped with Retinoic acid receptor activation (*p* = 8.71E-41), NFAT (*p* = 5.9E-43), and G-protein signaling through G-β/γ (*p* = 2.46E-38).

### Schizophrenia kinome network regulation

**Fig. 3:**
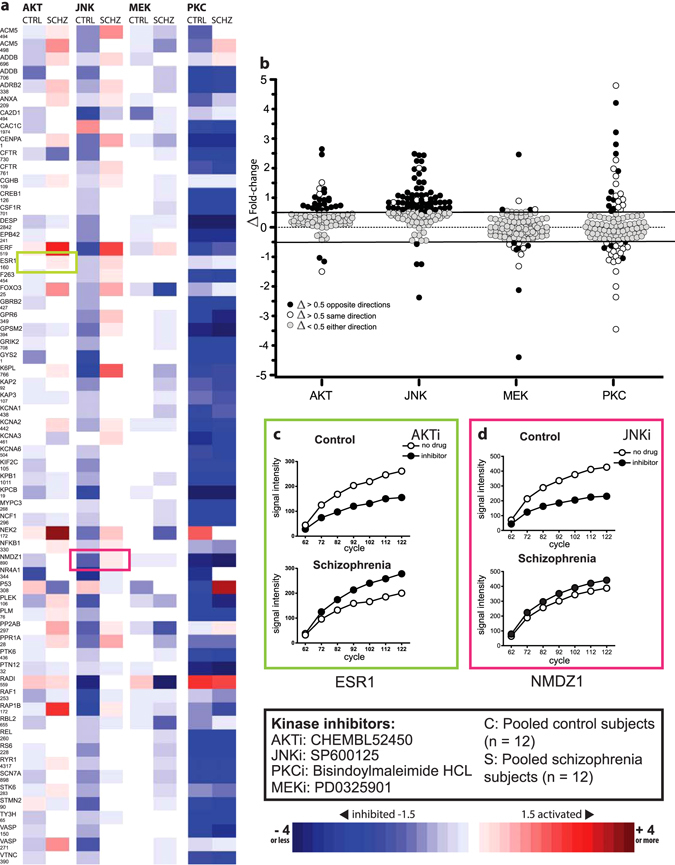
Serine/threonine Pamgene kinome array analysis of pooled control (C, *n* = 12) and schizophrenia (S, *n* = 12) run in the presence or absence of specific inhibitors (i) for AKT, JNK, MEK, and PKC. **a** Heat map showing the ratio of signal intensity of the sample with inhibitor/sample without inhibitor. *Lighter to darker blue* represents decreased phosphorylation (inhibition) on a specific array peptide, while *lighter to darker red* indicates increased phosphorylation (activation). Comparison of left and right sides in the column shows differential kinase activity and phosphorylation of peptide substrates between control and schizophrenia. **b** Differential phosphorylation by inhibitor type. *Black circles* represent peptide substrates with a difference in fold change of greater than 0.5, in which directionality (kinase activity increased, decreased, or not changed) was different between schizophrenia and control samples. *White circles* represent peptide substrates in which differences in fold change were greater than 0.5 but activity changed in the same direction in both samples. *Gray circles* represent peptide substrates in which the difference in fold change was less than 0.5 regardless of whether kinase activity was increased, decreased or unchanged on peptide substrates in both samples. **c**, **d** Representative enzyme kinetic curves for peptides substrates that were differentially phosphorylated by AKT (**c**) or JNK (**d**) inhibitors

To probe network regulation, we ran the kinome array with and without inhibitors targeting AKT, PKC, MEK, and JNK (Fig. 3a). We found AKT, PKC and MEK to be interconnected with schizophrenia-linked kinase “hits” (Fig. 2a), consistent with other studies implicating these kinases in schizophrenia.^9^ Additionally, we tested a JNK inhibitor due to its interactions with PKD and PAK, schizophrenia-linked kinase “hits” in our random sampling analysis.^10–12^

To increase stringency and include only substrates with kinase activity altered by the inhibitor compounds (rather than by intrinsic differences between the two samples), we increased our fold-change threshold to +/−1.5. By these criteria, the AKT inhibitor decreased kinase activity on four control substrates and two non-overlapping schizophrenia substrates, while increasing activity on six and zero substrates in the schizophrenia and control sample, respectively (Fig. 3a). The JNK inhibitor decreased kinase activity on 0 schizophrenia substrates and 29 substrates in the control sample (Fig. 3a). JNK inhibition increased kinase activity on one control and three schizophrenia substrates. MEK inhibition decreased kinase activity on two controls and six schizophrenia substrates (Fig. 3a). Finally, PKC inhibition decreased activity on 51 and 47 substrates in control and schizophrenia samples, respectively, and increased activity on two substrates in each sample (Fig. 3a).

We used the difference in fold-change (Δ fold-change) between schizophrenia and control to evaluate differential response to the kinase inhibitors (Fig. 3b). Substrates with a Δ fold-change of >0.5 were deemed to be differentially phosphorylated. JNK and AKT inhibitors showed the most divergence between schizophrenia and control (32.4% and 16.6% of substrates, respectively). These differences primarily reflect an inhibitor response in only one sample, or changes in opposite directions between samples (Fig. 3b, closed circles). PKC inhibitor robustly decreased kinase activity in both samples, but fewer substrates were differentially phosphorylated (9.4%); primarily these were differences in magnitude of change in the same direction (Fig. 3b, open circles). MEK inhibition produced the fewest differentially phosphorylated substrates (5%).

Representative examples of kinase activity for reporter peptides ESR1 (Fig. 3c) and NMDZ1 (Fig. 3d) highlight the differential effects of kinase inhibitors on the control and schizophrenia samples. Activity for ESR1 in decreased with the AKT inhibitor in the control sample, but increased with inhibitor in the schizophrenia sample (Fig. 3c). A similar pattern is observed for NMDZ1 (Fig. 3d) with the JNK inhibitor.

### Kinase proteins in schizophrenia

**Fig. 4:**
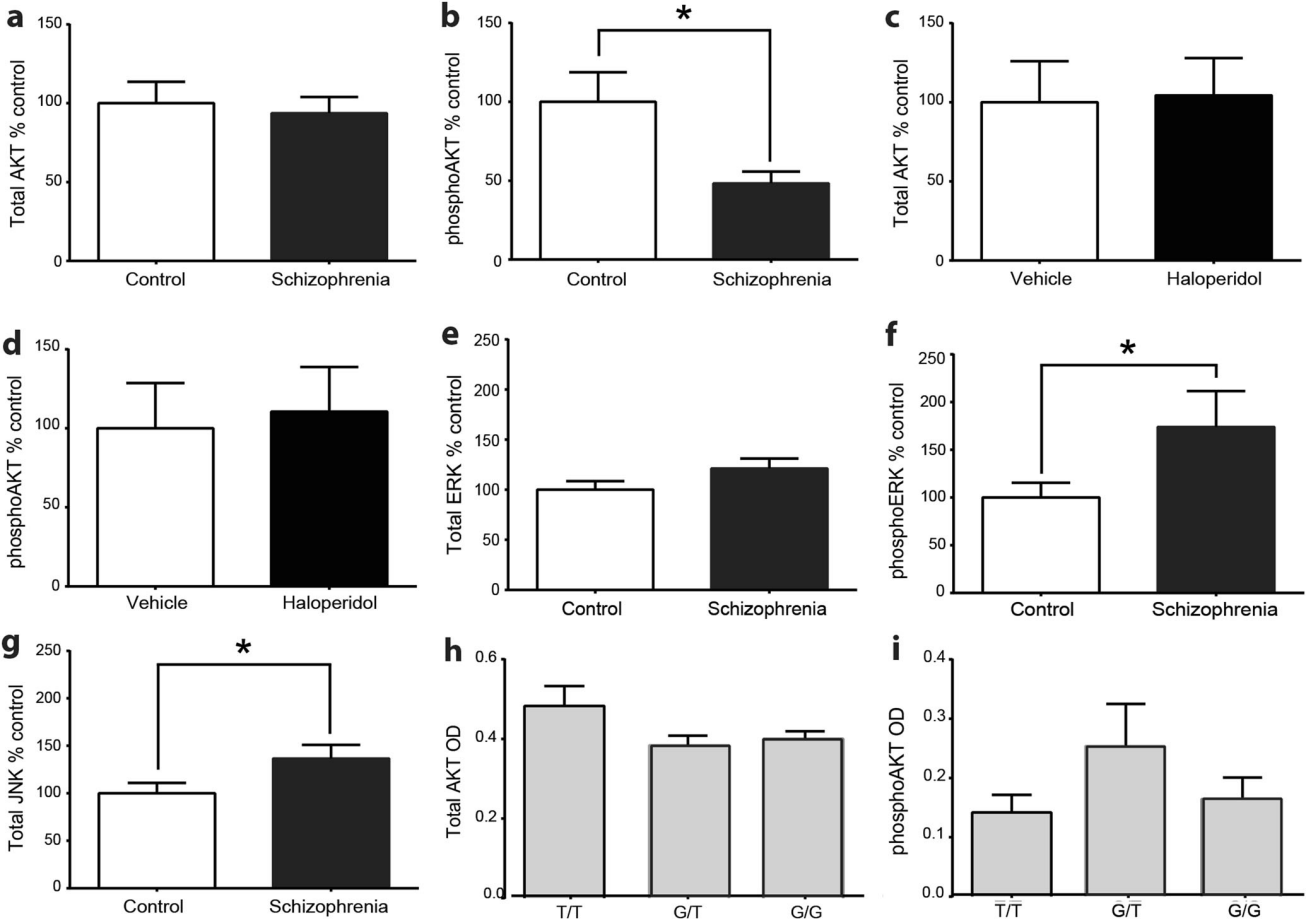
Total AKT (*p* = 0.43) and phosphoAKT (*p* = 0.038) in control versus schizophrenia ACC (**a**, **b**). Total AKT (*p* = 0.638) and phosphoAKT (*p* = 0.786) in vehicle versus haloperidol in rat (**c**, **d**). Total ERK (*p* = 0.097) and phosphoERK (*p* = 0.036) in control versus schizophrenia ACC (**e**, **f**). Total JNK (*p* = 0.023) in control versus schizophrenia ACC (**g**). Total AKT (*p* = 0.12) and phosphoAKT (*p* = 0.44) by genotype for the rs1130214 AKT SNP (**h**, **i**). *OD* optical density normalized to loading control. Welch’s *t*-test. Data expressed as mean +/−standard deviation; * indicates *p* < 0.05

We performed confirmation studies on targets identified from our bioinformatics analyses, using Western blot analysis to probe for differences in AKT, ERK1/2, or JNK protein or AKT and ERK1/2 phosphoprotein (Fig. 4 and Supplementary Fig. S1). Total AKT protein was similar between schizophrenia and control (*t* = 0.82; d*f* = 12; *p* = 0.43) (Fig. 4a), however phospho-AKT was decreased in schizophrenia subjects (*t* = 2.34; d*f* = 12; *p* = 0.038) (Fig. 4b and Supplementary Fig. S2). In contrast, haloperidol-treated and vehicle-treated rats had comparable total and phospho-AKT protein (*t* = 0.48; d*f* = 16; *p* = 0.638 and *t* = 0.28; d*f* = 16; *p* = 0.786, respectively) (Fig. 4c, d). Total ERK1/2 protein was unchanged between schizophrenia and control (*t* = 0.817; d*f* = 12; *p* = 0.097) (Fig. 4e), however phospho-ERK, driven by ERK1, was increased in schizophrenia (*t* = 2.27; d*f* = 12; *p* = 0.036) (Fig. 4f and Supplementary Fig. S2). Total JNK protein (*t* = 2.48; d*f* = 18; *p* = 0.023), was increased in schizophrenia (Fig. 4g). A breakdown of total and phosphoprotein levels by genotype for an AKT single nucleotide polymorphorphism (SNP), rs1130214, linked to schizophrenia identified no differences in the amounts of total or phosphoprotein for AKT (Fig. 4i, j).

### Kinase activity of AKT and JNK

**Fig. 5:**
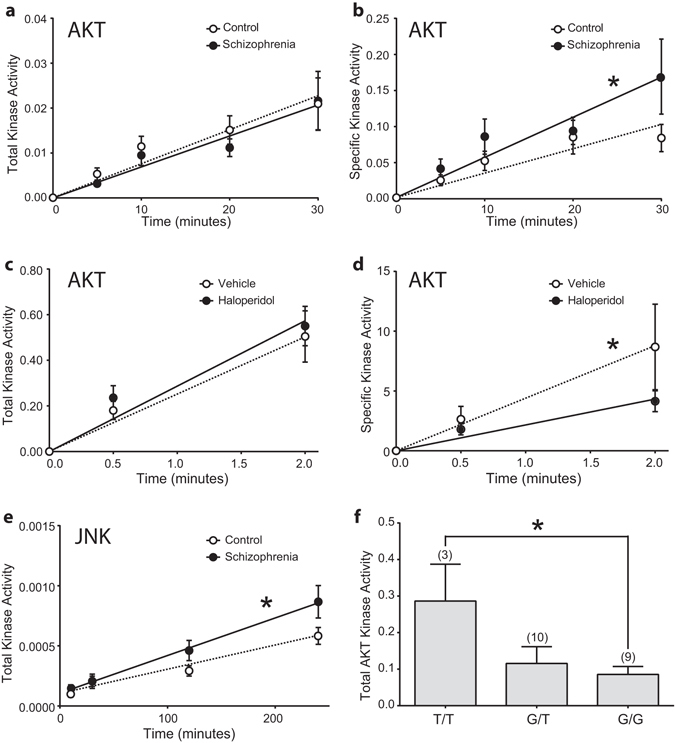
Enzyme activity for AKT and JNK kinase in schizophrenia and control subjects and haloperidol and vehicle-treated rats. Total AKT activity (**a**) specific AKT activity (**b**) in schizophrenia and control ACC. Total AKT activity (**c**) and specific AKT activity (**d**) in rat haloperidol and vehicle-treated frontal cortex. Total JNK activity (**e**) in schizophrenia and control ACC. Total AKT activity by rs1130214 SNP genotype (**f**) in ACC. Data are presented as mean +/−standard deviation; * indicates *p* < 0.05

Total AKT enzymatic activity was not different from control in schizophrenia (Fig. 5a) or haloperidol-treated rats (Fig. 5c) (*F* = 0.635(1,8); *p* = 0.449 and *F* = 0.276(1,8); *p* = 0.76, respectively). Specific activity, kinase activity relative to the amount of active kinase, was increased in schizophrenia (*F* = 11.99(1,8); *p* = 0.008) (Fig. 5c) and decreased in haloperidol-treated rats (*F* = 56.85(1,4); *p* = 0.002) (Fig. 5d). To confirm observed differences in AKT activity between schizophrenia and controls, we measured AKT activity in an additional group of eight matched schizophrenia and control pairs from the same brain bank (Supplementary Table S1 and Supplementary Fig. S3). As in the original cohort, no difference in total AKT activity was identified in the new subjects (Supplementary Fig. S3). AKT-specific activity was increased (54%) in schizophrenia in the enlarged data set of 20 subject pairs (*t* = 2.269, d*f* = 17, *p* = .0366) (Supplementary Fig. S3). Unexpectedly, despite increased JNK protein, total JNK enzymatic activity was decreased in schizophrenia (*F* = 5.46(2,84); *p* = 0.006) (Fig. 5e). No effects of age, sex, or postmortem interval (PMI) were found for the dependent measures.

### Analysis of SNP-dependent AKT activity

The original 12 subject pairs from the Mount Sinai repository were genotyped for AKT SNPs rs1130214 and rs2494732. These SNPs associate with schizophrenia as part of an AKT SNP haplotype.^13^ AKT activity was analyzed by SNP genotype irrespective of diagnosis. The rs1130214 minor allele is “T” and the allele distribution among the 24 subjects was G/G *n* = 9, T/G *n* = 12 and T/T *n* = 3. Total and phospho-AKT protein was unchanged across genotypes (F(2,21) = .857; *p* = 0.439 and F(2,19) = 2.36; *p* = 0.122) (Fig. 4i, j), however total activity (not shown) and specific activity (F(2,19) = 3.12; *p* = 0.042) were lower in G/G compared to T/T genotypes (Fig. 5f). The T/G genotype was intermediate to T/T and G/G genotypes in total and specific AKT activity consistent with the “T” allele at this location conferring increased AKT activity (Fig. 5f). In the second cohort, eight subjects were homozygous for the “G” allele and eight were heterozygous (T/G). As no subjects were homozygous T/T, we did not analyze the newer cohort for AKT activity by genotype. The major allele for rs2494732 is “T” and the allele distribution among the 40 subjects was T/T *n* = 10, C/T *n* = 18, C/C *n* = 11 and one undetermined. We found no differences between rs2484732 genotypes in AKT or phospho-AKT protein, or AKT activity (not shown).

## Discussion

Receptors, kinases, phosphatases, and other regulatory elements engage in complex, dynamic interactions as part of an intracellular signaling landscape that continuously responds to intra- and extracellular cues.^14^ Phenotypic similarities across the schizophrenia spectrum suggest that key components within the signaling milieu may be linked to core symptoms of schizo-spectrum disorders.^15^ Signal integration molecules, such as DARPP-32 and DISC1, were identified based on accumulations of individual observations^16^ and conceptualized as facilitators of “crosstalk” between unidirectional signaling cascades. Here, we identify a network of linked kinases based on a confluence of simultaneous independent observations. Functional analysis of this network aligns with cellular organization, molecular trafficking, and cell maturation suggesting compatible, perhaps simultaneous, alterations in dynamic cellular processes, such as structural organization and trafficking of organelles, vesicles, and macromolecules. These deficits may be catastrophic in inherently plastic systems such as activity-dependent remodeling of dendrites, spines, and synapses that need to rapidly adapt to and integrate environmental cues. Here, we expand on our previous findings by generating novel bioinformatics prediction algorithms to identify upstream kinases likely responsible for the abnormal kinase activity in the schizophrenia kinome.^4^

Our multi-step analysis identified kinases previously implicated in schizophrenia, including GRKs, CK, and PKA. Protein levels of GRKs are altered in cortical regions in schizophrenia,^17^ as are interactions between CK and PKA with DARPP-32.^18^ We also identified novel kinases not previously linked with schizophrenia, including DMPK, NEK, and PKD. Mutations in DMPK genes result in myotonic dystrophy, a syndrome involving muscle wasting, cardiac conductance defects, and endocrine defects.^19^ At least four DMPK splice variants are expressed in brain,^20^ and dysregulation affects both mRNA splicing and miRNA expression.^19^ DMPK is highly expressed in infancy, declining through childhood and early adulthood.^21^ Myotonic dystrophy frequently includes cognitive and neuropsychiatric symptoms similar to autistic spectrum disorders.^22^ NEKs are active in microtubule organization in the centrosome during cell division, however additional functions are likely as NEK expression is high in post-mitotic brain cells.^23^ Eleven NEK-family kinases are expressed in human brain with widely varying developmental profiles.^21^ PKD helps specify and maintain cell polarity by regulating sorting, packaging, and trafficking of membrane-associated proteins.^24^ PKD expression remains relatively stable, declining slightly through childhood and increasing again as the brain ages.^21^ Given the types of microstructural abnormalities described in schizophrenia,^25^ further investigation DMPK, NEK and PKD is warranted.

Kinomic analyses of prefrontal cortex from rats treated chronically with haloperidol decanoate produced a less complex kinase activity profile than that of our human samples. This profile reflects specific contributions of chronic antipsychotic treatment to changes in kinase activity, as these rats were not genetically modified, lesioned, or otherwise manipulated. The largest node in our haloperidol network analysis is AKT, which is supported in the literature.^26, 27^ Interestingly, AKT is also an important node in the schizophrenia network. PKA and DMPK were identified in both schizophrenia and haloperidol networks. However, the directionality of substrate phosphorylation (increase or decrease) in overlapping peptides between the human and rat data sets was opposite. Some overlap between the two networks is expected given that haloperidol is effective for some schizophrenia symptoms.^27^ Arguably, one would expect the overlapping findings to have opposite valences. These data suggest that chronic antipsychotic treatment does not explain all of our findings.

We performed additional kinome studies with inhibitors of kinases implicated (AKT, MEK, and PKC) and not strongly implicated (JNK) in schizophrenia. Although not well studied in schizophrenia, JNK dysregulation impacts neuronal architecture and plasticity.^28^ Differential phosphorylation of target substrates between schizophrenia and controls, with and without inhibitors, provides insight into upstream kinase network disruption. PKC inhibition decreased kinase activity in both samples and differentially phosphorylated only 9.4% of substrates (Fig. 3). Thus, we conclude that pan-inhibition of α, β, δ, ε, and γ PKC iso-enzymes with bisindolylmaleimide 1 failed to provide strong evidence of PKC dysregulation in schizophrenia. Inhibition of the ERK kinase MEK caused differential phosphorylation in only 5% of target substrates. Despite observed differences in phospho-ERK protein between schizophrenia and control, dysregulation of ERK signaling was not apparent, consistent with previous studies.^29^

Unexpectedly, AKT and JNK inhibitors produced the biggest differences in kinase activity between schizophrenia and control samples. Our data show that JNK and AKT inhibition induced differential phosphorylation in 45 (32.4%) and 23 peptide substrates (16.6%), respectively, between schizophrenia and control. These results suggest altered activity or regulation of these kinases. Serine–threonine kinases target phosphorylation sites through the chemical properties of flanking amino acid sequences, but will phosphorylate suboptimal sites, particularly when competition from other kinases is low and additional specificity factors are lacking.^14^ One hypothesis could be that changes in activity or specificity of AKT or JNK in schizophrenia allow other kinases to compete at sequences normally optimal for AKT or JNK. In this case, inhibitors of AKT or JNK would have minimal effect. These results should be interpreted cautiously as they are acquired using one concentration of a single inhibitor where phosphatase activity is inhibited, and mechanisms of target specificity may be disrupted. Although many aspects remain to be investigated, overall these data support widespread dysregulation in an interconnected signaling network including both AKT and JNK.

Using traditional biochemical tools, we identified differences in total and phosphoprotein expression; however this did not necessarily reflect kinase activity. Although total JNK protein was increased, JNK enzymatic activity was decreased in the schizophrenia samples, in agreement with data from our inhibitor study (Fig. 3). JNK activity is inhibited by interaction with PKD^11^ one of the kinases identified by random sampling analysis. In contrast, while phospho-AKT was decreased, AKT-specific activity was increased in schizophrenia samples (but not in haloperidol-treated rats).

We examined differences in AKT activity in two SNPs implicated in schizophrenia.^30^ rs1130214, but not rs2494732, appeared to correspond to AKT enzymatic activity. rs1130214 is a G to T intronic variant upstream of the 5′UTR.^31^ The substitution may play a role in regulating AKT transcription as it falls within an E2F transcription factor binding site and corresponded to reduced AKT protein levels in a previous schizophrenia cohort.^31^ We detected no differences in total AKT protein, or phosphoprotein, between SNP rs1130214 genotypes, however AKT activity was increased in homozygous carriers of the minor allele. A study of colorectal cancer subtypes also failed to identify changes in AKT expression with the rs1130214 SNP, however rs1130214 did associate, in this study, with reduced expression of pyruvate dehydrogenase kinase 1 (PDK1), a key component of the AKT signaling network.^32^ The “T” genotype of rs1130214 SNP is additionally associated with poor treatment response and increased morbidity in some types of cancer, presumably through AKT-mediated resistance to apoptosis and hyperactivity of pro-survival signaling networks.^33, 34^ However the mechanisms by which rs1130214 genotype may affect protein function are unknown. These data indicate that protein levels and phosphorylation states of kinases or target substrates used as proxy measurements of kinase activity may not reflect actual differences in enzyme activity.

The primary limitation of this study is the specificity of the substrates on the kinome array for specific kinases. Since kinase families have highly conserved catalytic and substrate recognition domains, so the peptides substrates on the array examined may have off-target signals. While we used Phosphonet and GPS 2.1 to make predictions based on sequence homology to known serine–threonine consensus sequences, many of these predictions are not experimentally verified. In addition, we only used one specific inhibitor at one concentration for each of the inhibitor studies. While our inhibitor studies with the kinome array provide striking evidence for differential regulation of signaling networks between schizophrenia and control samples, more work is needed to confirm the specificity of the pathways targeted.

There are other limitations to this study. Replication of these experiments in a larger population would strengthen the results and perhaps introduce new and pathophysiologically important components into the network. Additionally, due to the small initial sample size, the analysis of AKT activity by genotype is underpowered. Evaluation of atypical antipsychotics in the kinome array may provide additional insight into treatment effects on the signaling milieu. It is possible that unidentified kinases co-isolate with the kinases targeted in our activity studies, despite conditions designed to impede co-isolation. We also did not examine isoforms for AKT or ERK kinases, which have diverse functions.^35, 36^ Pathway analysis software such as Ingenuity relies on published findings and is therefore limited to information available in accessible databases (e.g., UniProt).^37^ Ingenuity is but one of several pathway analysis algorithms, each with inherent bias. A priority for understanding cell signaling dysregulation in schizophrenia should include evaluating phosphatase activity and the balance of phosphorylation/dephosphorylation within brain structures and cell types. Phosphatases including phosphatase and tensin homolog (PTEN), striatal-enriched protein tyrosine phosphatase, and the phosphoprotein phosphatase family are key contributors to plasticity and cognition.^38^

Our group and others have reported region-specific and cell-specific changes in gene expression in this illness.^39, 40^ Thus another limitation to this study is that we only examined a single brain region, the anterior cingulate cortex (ACC), at the region level, while changes in signaling networks could be region and cell-type specific^41, 42^ Although cell-level studies of kinase activity will be challenging in postmortem brain, they may be technically feasible and represent an important future direction for this work.

Overall, our approach provides a data set with higher relevance to the pathophysiology of schizophrenia because the analysis is independent of peptide substrate identity. Array substrates were selected based on their utility as reporter peptides rather than relevance to a disease state. Further, this unbiased approach better reflects the complexity of the kinome, as kinases have multiple overlapping targets, are degenerate with regard to specificity, and thus may be over or under-represented in a disease dependent manner.

These data argue for subtle changes in kinase activity and regulation across an interlinked kinase network. This is important as small changes in network dynamics may be amplified over multiple signaling events. Differences in kinase activity did not reflect changes in kinase protein or phosphoprotein levels. These results suggest signaling imbalances mediate compatible and likely simultaneous deficits in cellular processes underlying the core symptoms of schizophrenia.

In conclusion, to our knowledge no study has previously employed this kinome array platform in postmortem brain from subjects with schizophrenia, imputed the kinase assignments to the reporter peptides, used permutation analyses to identify kinases over or under represented in the data set, and built a kinase interaction model that reflects the complex nature of protein kinase interactions found in biological samples. Further, using a target of convenience identified from our hypothesis generating kinome array studies (AKT), for which well-characterized reagents are available, we confirmed alterations in AKT expression and activity in the same biological samples. It follows that the other nodes identified in our model are putative targets to investigate the pathophysiology of signaling defects in severe mental illness.

## Methods

### Overview

We performed four discrete experiments: (1) A bioinformatics analysis of our published kinome array data set, (2) studies on the impact of chronic haloperidol treatment on kinase activity (3) preliminary investigations using kinase inhibitors in the same array platform and subjects as experiment 1, and (4) biochemical studies for targets identified in experiments 1–3.

### Subjects

Fresh frozen postmortem (Brodmann area 24/32) was provided by the Mount Sinai NIH Brain and Tissue Repository (New York, NY). Schizophrenia (*n* = 12) and control samples (*n* = 12) were matched into pairs for age, sex, tissue pH, and PMI (Supplementary Table S1).^4^ A second cohort of non-overlapping schizophrenia (*n* = 8) and control (*n* = 8) ACC from the Mount Sinai repository, used to confirm changes in AKT activity, were similarly matched (Supplementary Table S1). Investigators were not blinded to subject group. Supplementary Table S1 provides demographics for the subjects included in these studies.

### Rodent studies

Adult male Sprague-Dawley rats (250 g) were housed in pairs and maintained on a 12 h light/dark cycle. Rats were randomly assigned to received 28.5 mg/kg haloperidol decanoate (*n* = 10) or vehicle (*n* = 10 sesame oil) by intramuscular injection every 3 weeks for 9 months.^39^ Rats were killed by rapid decapitation and brain tissue (prefrontal cortex) was dissected, flash frozen on dry ice, and stored at −80 °C until use. Investigators were not blinded to treatment condition. Sample size estimates were based on variability in previous biochemical studies using these tissues.^17^ All studies were performed in accordance with relevant guidelines and regulations and approved by the University of Alabama Birmingham Institutional Animal Care and Use Committee.

### PamGene kinome array

Kinase activity was previously assayed using the PamGene STK array.^4^ The array measures real-time kinase activity by monitoring phosphorylation of 139 distinct peptide substrates every 6 s over 90 min. In these initial studies, we validated the array in postmortem samples and showed that PMIs of 48 h (well beyond those of our postmortem samples) did not significantly impact kinase activity (4). The resulting data set provided a foundation for a novel bioinformatics workflow adapted from other proteomics studies.^43, 44^

### Identification of upstream kinases

Protein kinases targeting 19 previously identified peptide substrates were assigned using Kinexus Phosphonet (Kinexus Bioinformatics) and GPS 2.1 algorithms.^45, 46^ The three top-ranked kinases predicted at each phosphorylation site in Phosphonet and kinases with scores 2 or more times the prediction threshold at each site in GPS 2.1 were included as predicted kinases. By using two prediction algorithms we attempted to minimize prediction biases inherent in each one.

### Random sampling analyses

We identified key kinases in the schizophrenia kinome using random sampling analysis.^44^ We generated 2000 data points composed of 19 randomly selected reporter peptide substrates from 139 possibilities on the array. Using Phosphonet and GPS 2.1, we assigned kinases to every substrate, calculated the frequency of kinases for each datapoint, and generated “expected” distributions for each kinase. Means and standard deviations were calculated for each distribution and kinases with observed frequencies beyond two standard deviations from the expected mean were carried forward into our network analyses.

### Network modeling

From overrepresented kinases identified by random sampling, we generated a network model beginning with direct interactions between the kinases. The network was expanded by restricting the IPA grow tool to “kinases” and “direct interactions”.^43^ The emerging network was refined by removing kinases with fewer than two connections. Since the number of interactions between kinases may determine relative importance of a kinase to the network, we weighted our model using the number of interactions for each kinase in the network.^47^

### Ingenuity pathway analyses (IPA)

The resulting kinase networks were analyzed using IPA for associated functions and canonical pathways.^4^

### Exploratory kinome array studies

10 ug frontal cortical homogenate from haloperidol (*n* = 10) and vehicle (*n* = 10)-treated rats were pooled to make one haloperidol and one vehicle sample. Kinome arrays were performed and fold-change was calculated from the ratio of haloperidol/control kinase activity for each substrate.^4^ Substrates with a fold-change +/−1.15 or greater in kinase activity underwent random sampling and network analyses.

10 ug ACC homogenate from schizophrenia (*n* = 12) and control (*n* = 12) subjects were pooled to make one schizophrenia and one control sample. Samples were evaluated by kinome array with or without inhibitors for AKT (Calbiochem 124005), JNK (SP600125, Calbiochem), MEK (D-erythro-sphingosine N-hexanoyl Calbiochem), or PKC (Bisindoylmaleimide Hydrochloride, Cell Signaling) at a concentration of 150 uM.^4^ Fold-change was calculated from the ratio of kinase activity (inhibitor/no inhibitor). Differences in fold-change (Δ fold-change) were calculated as (schizophrenia + inhibitor/schizophrenia no inhibitor)—(control + inhibitor/control no inhibitor). Substrates without detectable signal in the inhibitor-free sample were excluded.

### Biochemical studies

Biochemical studies utilized unpooled ACC samples from the original 12 subject pairs and, for AKT enzyme activity and 8 additional subject pairs from the same repository.

### Western blot analyses

Membranes were incubated overnight at 4 °C in primary antibodies, including rabbit AKT (1:250 dilution, Cell Signaling 9272), mouse p44/42 MAPK (ERK1/2) (1:250, Cell Signaling 4696), rabbit phospho-p44/42 (ERK1/2)(1:1000, Cell Signaling 4370), and mouse anti-valosin-containing protein (1:4000, Abcam). Phosphorylated AKT was determined by capturing phospho-AKT on sepharose beads using phospho-AKT(S473) 1:100 (Cell Signaling 4070) and probing for AKT using rabbit anti-AKT 1:1000 (Cell Signaling 9272). Phospho-AKT protein was normalized to immunoglobulin heavy and light chains from the sepharose beads. LiCor secondary antibodies raised against the appropriate species were used at 1:5000 as previously described.^48^ Representative blots are shown in Supplementary Fig. S1. Blots were processed in parallel.

### Kinase activity assays

For the AKT assay (Cell Signaling, #9840), 120 µg of homogenate was combined with 120 µl anti-phosphoAKT bound sepharose beads and sterile H2O for a reaction volume of 1320 µl, and rotated overnight at 4 °C. The beads were pelleted by centrifugation at 4 °C and the supernatant was discarded. The beads were resuspended in kinase buffer and equally divided into tubes for each time point (0, 5, 10, and 30 min) and a negative control (no ATP). 2 µl GSK3 fusion protein and 0.8 µl ATP was added to each and the samples were maintained at 30 °C. 3x Blue Loading Buffer supplemented with DTT (Cell Signaling #7722 S) terminated the reaction. The sepharose beads were pelleted by centrifugation and the supernatant was retained. Kinase activity was quantified by western blot from phosphorylated GSK3a/b fusion protein levels normalized to total GSK3 fusion protein. Specific AKT kinase activity was determined by normalizing to the amount of phosphoAKT protein captured by the beads. The JNK assay (Cell Signaling, #8794) was performed with the following modifications to the above protocol. An amount of 180 µg of homogenate was combined with 180 µl beads and sterile H2O for a reaction volume of 1380 µl. Samples were divided equally into 6 tubes for a negative control and time points at 0, 10, 30, 120, 240 min. A volume of 2 µl cJun fusion protein and 2 µl ATP (except negative control) were added and the samples were incubated at 30 °C. Once the reaction was terminated, the beads were pelleted by centrifugation and the supernatant was analyzed by western blot for the phosphorylated reporter protein.

### SNP assays

ACC samples were genotyped for AKT SNPs with Taqman genotyping assays C_26352825_10 (rs1130214) and C_16191608 (rs2494732).^39^

### Statistical analyses

Data were analyzed with Statistica (Statsoft Inc) and/or Prism Graphpad (Graphpad Software Inc). Kinase frequency distributions were tested for normalcy using D’Angostine and Pearson omnibus normality test. Ingenuity calculates significance using the right-tailed Fisher Exact Test. Associations were tested between the dependent measures and age, tissue pH, or PMI by multiple regressions. Western blot and AKT SNP allele data were tested for outliers in Prism using robust regression outlier removal with a Q coefficient of 5%,^49^ for normalcy using D’Angostine and Pearson, and analyzed using Welch’s unpaired two-tailed t-test or one-way ANOVA with Sidak’s correction for multiple comparison. Data not meeting normalcy criteria we analyzed using the nonparametric Mann–Whitney test. Slopes of the best-fit lines for kinase activity were analyzed using regression analyses. Data is presented as mean +/− standard deviation. For all tests alpha was 0.05.

### Data sharing

The data sets generated during and/or analyzed during the current study are available from the corresponding author on reasonable request.

## Electronic supplementary material

Supplemental Material
