# Supplementary material for: Abnormalities of signal transduction networks in chronic schizophrenia
Source: NPJ Schizophr. 2017 Sep 12;3:30. doi: 10.1038/s41537-017-0032-6 (PMC5595970; doi:10.1038/s41537-017-0032-6)
Supplement: Supplementary file 1 — Supplemental Material [file 41537_2017_32_MOESM1_ESM.docx]

Supplemental Table 1. Demographics of original 12 schizophrenia and 12 control and additional 8 schizophrenia and 8 control subject pairs

| Diagnosis | Sex | Age (y) | Medication | PMI (m) | pH | Rs1130214 | Rs2494732 |
| --- | --- | --- | --- | --- | --- | --- | --- |
| Control | F | 74 | no antipsychotic | 180 | 6.0 | T/G | C/C |
| Schizophrenia | F | 74 | haloperidol | 417 | 6.3 | T/G | T/T |
| Control | F | 84 | no antipsychotic | 1110 | 6.21 | T/T | C/C |
| Schizophrenia | F | 86 | haloperidol | 1092 | 5.8 | T/G | C/T |
| Control | F | 78 | no antipsychotic | 600 | 6.19 | T/G | C/T |
| Schizophrenia | F | 77 | risperidone | 583 | 6.01 | G/G | T/T |
| Control | F | 80 | no antipsychotic | 285 | 6.2 | G/G | C/T |
| Schizophrenia | F | 80 | thiothixene, chlorpromazine | 415 | 5.8 | T/G | C/T |
| Control | M | 95 | no antipsychotic | 245 | 6.53 | T/G | C/T |
| Schizophrenia | M | 97 | haloperidol | 555 | 6.5 | T/T | C/C |
| Control | M | 59 | no antipsychotic | 1225 | 6.63 | G/G | C/T |
| Schizophrenia | M | 57 | unknown | 1220 | 6.1 | T/G | C/C |
| Control | F | 88 | no antipsychotic | 305 | 6.4 | G/G | T/T |
| Schizophrenia | F | 90 | haloperidol, olanzapine | 465 | 5.97 | G/G | C/T |
| Control | F | 66 | no antipsychotic | 960 | 6.51 | G/G | C/T |
| Schizophrenia | F | 69 | thioridazine | 820 | 6.2 | G/G | C/C |
| Control | M | 69 | no antipsychotic | 255 | 6.3 | T/T | C/T |
| Schizophrenia | M | 68 | no antipsychotic* | 534 | 6.27 | T/G | C/T |
| Control | M | 65 | no antipsychotic | 230 | 6.82 | G/G | T/T |
| Schizophrenia | M | 63 | haloperidol | 372 | 5.9 | T/G | C/C |
| Control | M | 72 | no antipsychotic | 720 | 6.6 | T/G | T/T |
| Schizophrenia | M | 73 | haloperidol  fluphenazine | 475 | 6.5 | T/G | T/T |
| Control | M | 66 | no antipsychotic | 454 | 6.58 | T/G | C/T |
| Schizophrenia | M | 66 | no antipsychotic* | 725 | 6.5 | G/G | C/C |

| Diagnosis | Sex | Age (y) | Medication | PMI (m) | pH | Rs1130214 | Rs2494732 |
| --- | --- | --- | --- | --- | --- | --- | --- |
| Control  Schizophrenia | F  F | 79  81 | no antipsychotic  risperidone  olanzapine | 431  750 | 5.72  5.93 | T/G  T/G | T/T  C/C |
| Control  Schizophrenia | F  F | 86  82 | no antipsychotic  haloperidol  olanzapine | 610  530 | N/A  5.89 | T/G  G/G | C/T  C/T |
| Control  Schizophrenia | M  M | 64  70 | no antipsychotic  haloperidol | 624  855 | 6.12  6.49 | T/G  T/G | C/T  C/C |
| Control  Schizophrenia | M  M | 70  70 | no antipsychotic  thiothixene  olanzapine | 1430  1038 | 6.04  6.36 | G/G  G/G | C/C  C/T |
| Control  Schizophrenia | M  M | 71  73 | no antipsychotic  no antipsychotic* | 1285  430 | N/A  6.35 | G/G  T/G | C/T  C/T |
| Control  Schizophrenia | M  M | 73  73 | no antipsychotic  risperidone | 1264  525 | 6.94  6.15 | G/G  G/G | T/T  C/T |
| Control  Schizophrenia | M  M | 78  75 | no antipsychotic  no antipsychotic* | 485  345 | N/A  5.85 | G/G  T/G | T/T  U |
| Control  Schizophrenia | M  M | 79  77 | no antipsychotic  risperidone,  olanzapine | 964  1440 | N/A  6.4 | G/G  T/G | T/T  C/C |

Abbreviations: Female (F), Male (M), years (y), minutes (m), Postmortem Interval (PMI), not available (N/A), undetermined “U”. Rs1130214 and Rs2494732 are single nucleotide polymorphisms (SNPs) of protein kinase B (AKT), G/G, T/G, T/T C/T, and C/C are the genotypes for each SNP. *indicates off all antipsychotic medications within 6 weeks of death.

Supplemental table 2. 19 peptide substrates differentially phosphorylated in schizophrenia versus control subjects on the Pamgene kinome array

| Peptide  Increased | Fold  Change | Abbreviation | Uniprot  Accession | | | |
| --- | --- | --- | --- | --- | --- | --- |
|  |  |  |  |  | | |
| G-protein signaling modulator 2 | 1.329 | GPSM2 |  | P81274 | | |
| Phosphorylase b kinase alpha M subunit | 1.295 | PHKA1 |  | P46020 | | |
| Cystic fibrosis transmembrane conductance regulator | 1.263 | CFTR |  | P13569 | | |
| β-2 adrenergic receptor | 1.250 | ADRB2 |  | P07550 | |  |
| cAMP-responsive element-binding protein | 1.221 | CREB1 |  | P16220 | |  |
| Macrophage colony-stimulating  factor 1 receptor | 1.217 | CSF1R |  | P07333 | |  |
| Annexin 1 | 1.204 | ANXA1 |  | P04083 | |  |
| Vasodilator-stimulated  phosphoprotein | 1.192 | VASP |  | P50552 | |  |
| Erythrocyte protein 4.2 | 1.185 | EPB42 |  | P16452 | |  |
| Protein tyrosine kinase 6 | 1.180 | PTK6 |  | Q13882 | |  |
| G-protein coupled receptor 6 | 1.164 | GPR6 |  | P46095 | |  |
| Phospholemman ion transport regulator | 1.156 | FXYD1 |  | O00168 | |  |
| Proto-oncogene c-Rel | 1.152 | REL |  | Q04864 | |  |
| Vitronectin adhesion molecule | 1.152 | VTN |  | P04004 | |  |
|  |  |  |  |  | |  |
| Peptide  Decreased | Fold Change | Abbreviation | Uniprot  Accession | |  |  |
|  |  |  |  |  | |  |
| Ets-domain containing protein | -1.446 | ELK1 |  | P19419 | |  |
| Myelin basic protein | -1.300 | MBP |  | P02686 | |  |
| E3 ubiquitin ligase | -1.277 | CBL |  | P22681 | |  |
| Serine/threonine protein kinase 2 | -1.242 | CHEK2 |  | O96017 | |  |
| Tyrosine protein kinase | -1.173 | ZAP70 |  | P43403 | |  |

14 peptides exhibited a 1.15-fold or more increase in kinase activity while 5 exhibited a 1.15-fold or more decrease in phosphorylation.

Supplemental table 3. 16 peptide substrates differentially phosphorylated in rats treated chronically with haloperidol versus untreated rats on the Pamgene kinome array

| Peptide  Increased | Fold  Change | Abbreviation |  | Uniprot  Accession | |
| --- | --- | --- | --- | --- | --- |
| NIMA-related kinase 3 | 1.18 | NEK3 | |  | P51956 |
|  |  |  | |  |  |
| Peptide  Decreased | Fold Change | Abbreviation | | Uniprot  Accession | |
| Raf-1 proto-oncogene | -1.625 | RAF1 | |  | P04049 |
| CD27 molecule | -1.501 | CD27 | |  | P26842 |
| Cystic fibrosis transmembrane conductance regulator (ATP-binding cassette sub-family C, member 7) | -1.371 | CFTR | |  | P13569 |
| RAP1B, member of RAS oncogene family | -1.305 | RAP1B | |  | P61224 |
| Protein kinase C, beta | -1.287 | PRKCB | |  | P05771-2 |
| Vasodilator-stimulated phosphoprotein | -1.283 | VASP | |  | P50552 |
| Neutrophil cytosolic factor 1 | -1.252 | NCF1 | |  | P14598 |
| Stathmin 2 | -1.230 | STMN2 | |  | Q93045 |
| Annexin A1 | -1.222 | ANXA1 | |  | P04083 |
| Glycogen synthase 2 (liver) | -1.216 | GYS2 | |  | P54840 |
| histone cluster 1, H2bb | -1.213 | HIST1H2BB | |  | P33778 |
| microtubule-associated protein tau | -1.206 | MAPT | |  | P10636 |
| v-rel avian reticuloendotheliosis viral oncogene homolog | -1.777 | REL | |  | Q04864 |
| tyrosine hydroxylase | -1.161 | TH | |  | P07101 |
| vitronectin | -1.161 | VTN | |  | P04004 |

1 peptide exhibited a 1.15-fold or more increase in kinase activity while 15 exhibited a 1.15-fold decrease in phosphorylation.

Supplemental Table 4. Predicted kinases and distributions for schizophrenia and haloperidol datasets

| Schizophrenia | | | | | |
| --- | --- | --- | --- | --- | --- |
| Kinase | Observed Hits | Distribution Mean | Standard Deviation | Z-score | Confidence Interval |
| Confidence Interval ≥ 2 STD | | | | | |
| PAK | 12 | 3.60 | 1.63 | 5.15 | 0.34 to 6.85 |
| PKD | 5 | 1.62 | 1.13 | 2.99 | -0.64 to 3.88 |
| GRK | 13 | 7.03 | 2.00 | 2.99 | 3.03 to 11.02 |
| DMPK | 10 | 5.17 | 1.90 | 2.54 | 1.37 to 8.97 |
| CK | 7 | 3.29 | 1.50 | 2.47 | 0.28 to 6.30 |
| PKA | 12 | 7.20 | 1.95 | 2.46 | 3.29 to 11.10 |
| NEK | 3 | 0.88 | 0.87 | 2.44 | -0.87 to 2.62 |
| Confidence Interval between 1.5-2.0 STD | | | | | |
| PKC | 12 | 8.09 | 2.01 | 1.95 | 5.07 to 11.12 |
| CHK1 | 1 | 4.04 | 1.68 | -1.81 | 1.52 to 6.55 |
| CAMK4 | 4 | 1.91 | 1.22 | 1.71 | 0.09 to 3.74 |
| AKT | 7 | 4.37 | 1.71 | 1.54 | 1.81 to 6.92 |
| JNK | 6 | 9.04 | 2.01 | -1.51 | 6.02 to 12.05 |
| PKG | 8 | 5.20 | 1.86 | 1.51 | 2.41 to 7.99 |
| SGK | 5 | 2.81 | 1.46 | 1.50 | 0.62 to 4.99 |
| Haloperidol | | | | | |
| Kinase | Observed Hits | Distribution Mean | Standard Deviation | Z-score | Confidence Interval |
| Confidence Interval ≥ 2 STD | | | | | |
| AKT | 10 | 3.65 | 1.59 | 3.99 | 0.46 to 6.84 |
| PKA | 12 | 6.11 | 1.87 | 3.15 | 2.36 to 9.85 |
| DMPK | 8 | 4.31 | 1.66 | 2.22 | 0.99 to 7.64 |
| Confidence Interval between 1.5-2.0 STD | | | | | |
| CDK | 5 | 8.43 | 1.88 | -1.82 | 5.61 to 11.25 |
| RSK | 11 | 7.56 | 1.90 | 1.81 | 4.71 to 10.41 |
| p38 | 3 | 6.10 | 1.77 | -1.75 | 3.44 to 8.76 |
| ILK | 1 | 0.23 | 0.44 | 1.75 | -0.44 to 0.89 |
| PKG | 7 | 4.27 | 1.66 | 1.64 | 1.77 to 6.77 |
| CAMK2 | 8 | 5.19 | 1.73 | 1.62 | 2.60 to 7.79 |
| CHK1 | 1 | 3.39 | 1.52 | -1.57 | 1.11 to 5.66 |
| PRKX | 3 | 1.33 | 1.06 | 1.58 | -0.26 to 2.92 |

Abbreviations: Calcium/calmodulin-dependent protein kinase type 4 (CAMK4); calcium/calmodulin-dependent protein kinase type 2 (CAMK2); G-protein coupled receptor kinase (GRK); p21-activated kinase (PAK); protein kinase B (AKT); protein kinase D (PKD); casein kinase (CK); dystrophia myotonica-protein kinase (DMPK); never in mitosis gene A-related kinase (NEK); mechanistic target of rapamycin (mTOR); death-associated protein kinase (DAPK); protein kinase A (PKA); protein kinase G (PKG); protein kinase C (PKC); protein kinase, X-linked (PRKX); cyclin-dependent kinase (CDK); checkpoint kinase 1 (CHK1); c-Jun N-terminal kinase (JNK); serine/threonine-protein kinase (SGK); ribosomal s6 kinase (RSK); p38 mitogen-activated protein kinase (p38); integrin-linked kinase (ILK); standard deviation (STD).

Supplemental Table 5. IPA analyses

| Schizophrenia (Molecular and cellular functions associated with kinase networks) | | |
| --- | --- | --- |
| Functions | p-value | Associated kinases |
| Phosphorylation of protein | 1.99E-57 | GRK; CDK; CK; DMPK; LRRK2; JNK; PAK; PKA; PKC; RAF; CAMKK2; EGFR; MEK; ERK; PDK1; SRC; AKT; GSK3; p38; TGFBR |
| Organization of cytoplasm | 1.10E-25 | GRK; CK; GSK3; JNK; p38; PAK; PDK1; PKA; PKC; SRC; AKT; LRRK2; NEK; PKD;TBK1; DMPK; EGFR; MEK; ERK; PLK1; TGFBR; CDK; GIT1; RAF |
| Organization of cytoskeleton | 2.45E-24 | GRK; CK; GSK3; JNK; p38; PAK; PDK1; PKC; PKD; TGFBR; AKT;LRRK2; NEK; RAF; DMPK; EGFR; MEK; ERK; PKA; SRC; CDK; GIT1; TBK1 |
| Microtubule dynamics | 2.47E-20 | AKT; DMPK; CDK5; CK; EGFR; GIT1; GSK3; LRRK2; MEK; ERK; p38; JNK; NEK; PAK; PKA; PKC; SRC; TGFBR; RAF; TBK1 |
| Neurite growth | 6.13E-17 | AKT; CDK; CK; EGFR; GSK3; LRRK2; MEK; ERK; JNK; PAK; PKA; PKC; RAF; SRC |
| Neuronal differentiation | 3.7E-10 | AKT; ATM; CDK; EGFR; GSK3; MEK; ERK; p38; JNK; PAK; PKC; SRC |
| Long-term potentiation | 5.57E-09 | CAMKK2; EGFR; GSK3; MEK; ERK; PAK; PKA |
| Haloperidol (Molecular and cellular functions associated with kinase networks) | | |
| Phosphorylation of protein | 1.36E-38 | AKT; DMPK; GSK3; IKK; LRRK2; INSR; JNK; PDK1; PI3K; PKA; PKC; RAF; RIPK; SYK; SRC |
| cell viability | 9.06E-26 | AKT; DMPK; GSK3; IKK; INSR; LRRK2; JNK; PDK1; PI3K; PKA; PKC; RAF; RIPK; SRC; SYK; TBK1 |
| Cell viability of B lymphocytes | 3.48E-17 | AKT; IKK; PDK1; PI3K; PKC; SYK |
| Platelet aggregation | 9.25E-16 | AKT; GSK3; PDK1; PI3K; PKA; PKC; SYK |
| Differentiation of cells | 1.57E-15 | AKT; GSK3; IKK; LRRK2; JNK; PDK1; PI3K; PKA; PKC; RAF; RIPK; SRC; SYK |
| Proliferation of immune cells | 4.55E-14 | AKT; GSK3; IKK; JNK; PI3K; PKA; PKC; SRC; SYK |
| Inflammatory response | 2.83E-13 | AKT; IKK; LRRK2; JNK; PI3K; PKA; PKC; SRC; SYK; TBK1 |
| Cell movement of phagocytes | 6.92E-13 | AKT; IKK; LRRK2; JNK; PI3K; PKA; SYK |
| Schizophrenia (Canonical pathways) | | |
| Canonical signaling | p-value | Associated kinases |
| ErbB signaling | 3.47E-43 | AKT; ATM; EGFR; GSK3; MEK; ERK; JNK; p38; PAK; PDK1; PKC; PKD; RAF |
| Molecular mechanisms of cancer | 4.31E-41 | AKT; ATM; CDK; GSK3; MEK; ERK; JNK; p38; PAK; PKA; PKC; PKD; RAF; SRC; TGFBR |
| GNRH signaling | 1.94E-38 | EGFR; MEK; ERK; JNK; p38; PAK; PKA; PKC; PKD; RAF; SRC |
| Renin angiotensin signaling | 3.62E-38 | ATM; MEK; ERK; JNK; p38; PAK; PKA; PKC; PKD; RAF |
| Pyroxidol 5’-phosphate salvage | 5.55E-37 | AKT; CDK; CK; DMPK; MEK; ERK; JNK; NEK; PAK; PLK1; PKC |
| Haloperidol (Canonical pathways) | | |
| NFAT in cardiac hypertrophy | 5.9E-43 | AKT; GSK3; JNK; PI3K; PKA; PKC; RAF; SRC |
| RAR activation | 8.71E-41 | AKT; JNK; PDK1; PI3K; PKA; PKC; SRC |
| Gβγ signaling | 2.46E-38 | AKT; PDK1; PI3K; PKA; PKC; RAF; SRC |
| P2Y purigenic signaling | 8.19E-38 | AKT; RAF; PKC; PKA; PI3K |
| Gap junction signaling | 1.69 E-37 | AKT; PI3K; PKA; PKC; RAF; SRC |

Ingenuity Pathway results for schizophrenia and haloperidol network kinases ranked by p-value.

Supplemental Table 6. Kinase network regulation

| Schizophrenia (Kinase network regulation) | | | |
| --- | --- | --- | --- |
| Name | Abbreviation | p-value |  |
| Tumor protein 53 | p53 | 2.94E-14 | Apoptosis; senescence; DNA repair; changes in metabolism |
| Amyloid precursor protein | APP | 3.76E-14 | axon cargo transport; cell adhesion; apoptosis; platelet activation |
| Src proto-oncogene | Src | 2.5E-11 | cell-cell adhesion; cell cycle; cell migration; proliferation |
| Glucocorticoid receptor α 2 | NR3C1 | 1.44E-09 | inflammatory responses; proliferation; differentiation |
| Harvey rat sarcoma viral oncogene | HRAS | 2.61E-09 | Actin cytoskeletal organization, apoptosis; senescence; |
| Phosphatase and tensin homolog | PTEN | 1.52E-08 | Apoptosis; myelin maintenance; spine morphogenesis; angiogenesis |
| Epidermal growth factor | EGF | 4.81E-08 | Angiogenesis; coagulation; migration; ERK signaling |
| Haloperidol (Kinase network regulation) | | | |
| Glucocorticoid receptor α2 | NR3C1 | 1.53E-13 | inflammatory responses; proliferation; differentiation |
| proinsulin | Ins1 | 3.79E-09 | chaperone binding; hormone activity |
| Microtubule associated protein 2 | MAP2 | 5.40E-09 | microtubule assembly |
| Tripartite motif containing 41 | TRIM41 | 5.4E-09 | Ubiquitination; protein kinase C signaling |
| Tumor protein 53 | TP53 | 4.17E-08 | Apoptosis; senescence; DNA repair; changes in metabolism |
| Amyloid β precursor protein | APP | 4.44E-08 | axon cargo transport; cell adhesion; apoptosis; platelet activation |
| SRC proto-oncogene | SRC | 5.63E-08 | cell-cell adhesion; cell cycle; cell migration; proliferation |
| Growth factor independent 1 transcription repressor | GFI1 | 6.77E-07 | Histone modification; transcription regulation |
| Neural retina leucine zipper | NRL | 1.52E-06 | Transcription regulation |
| phospholamban | PLN | 3.09E-06 | calcium ion transport; ATPase inhibitor activity |

**
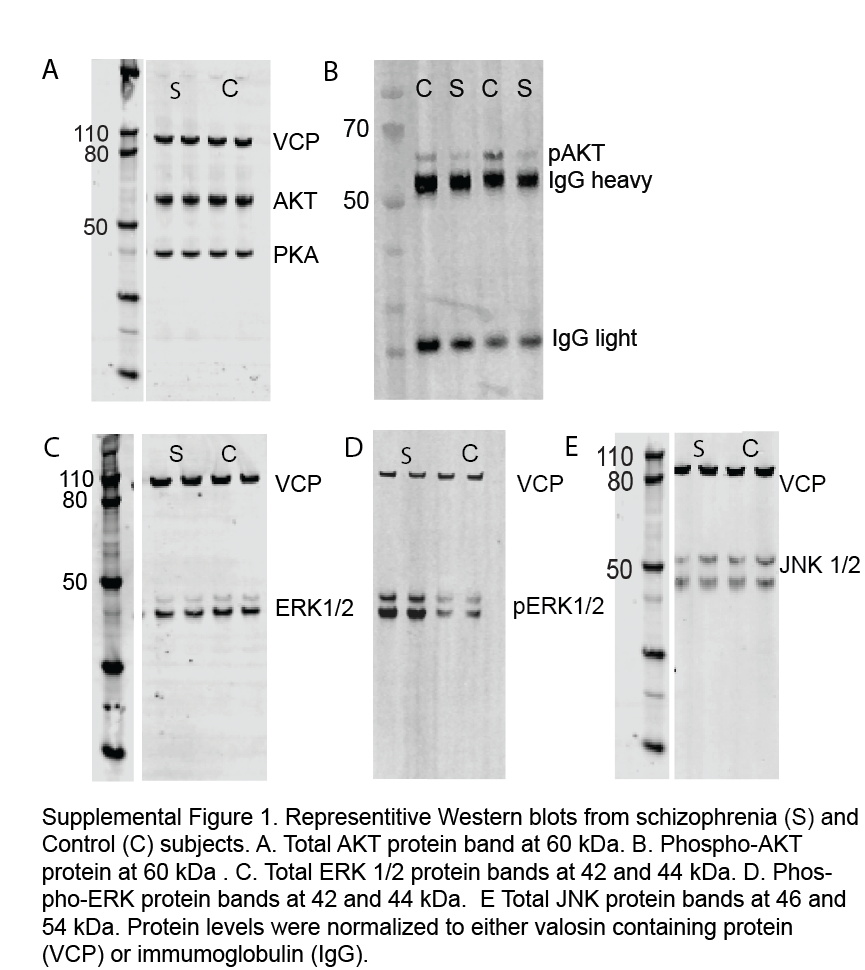
**

**
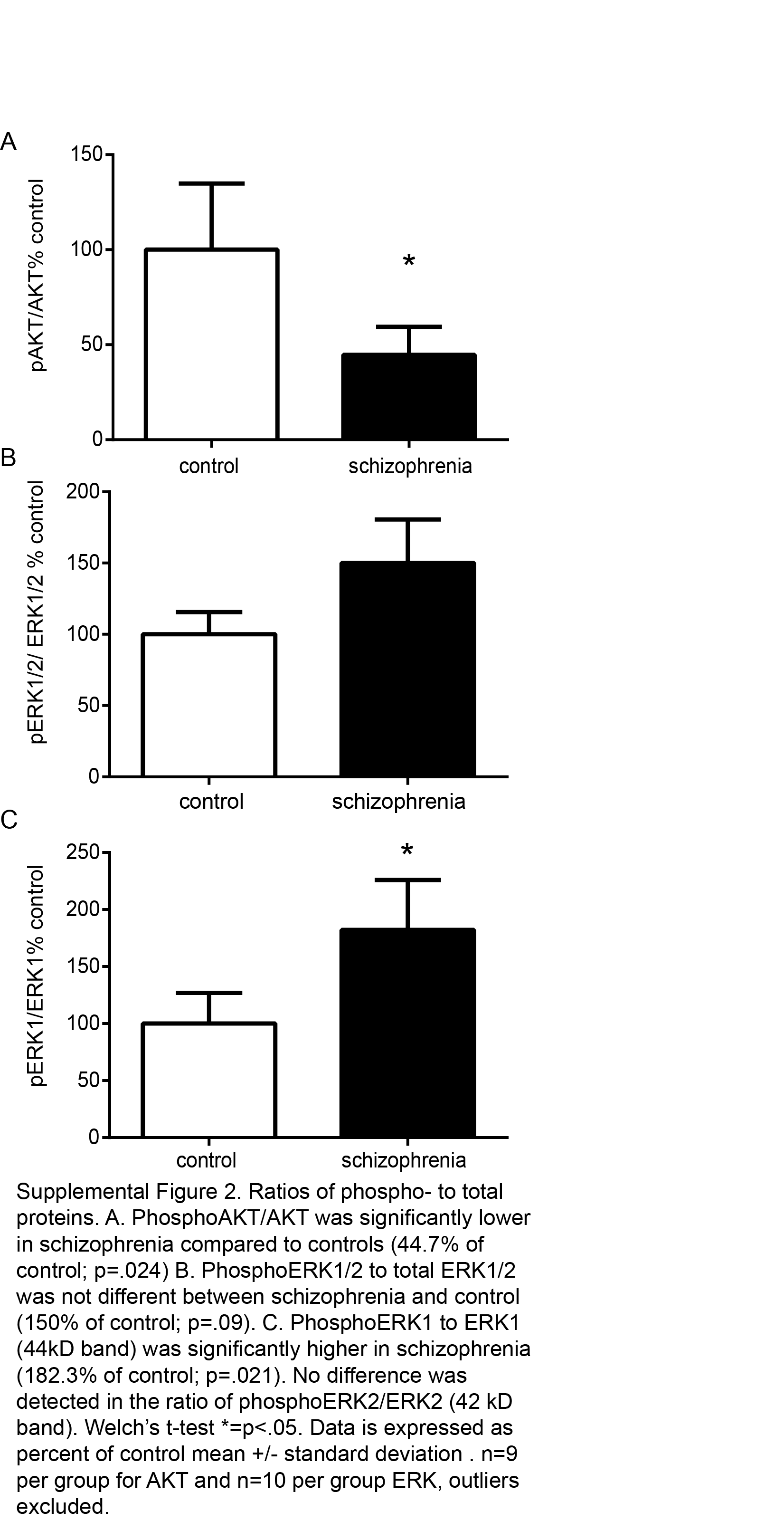
**

**
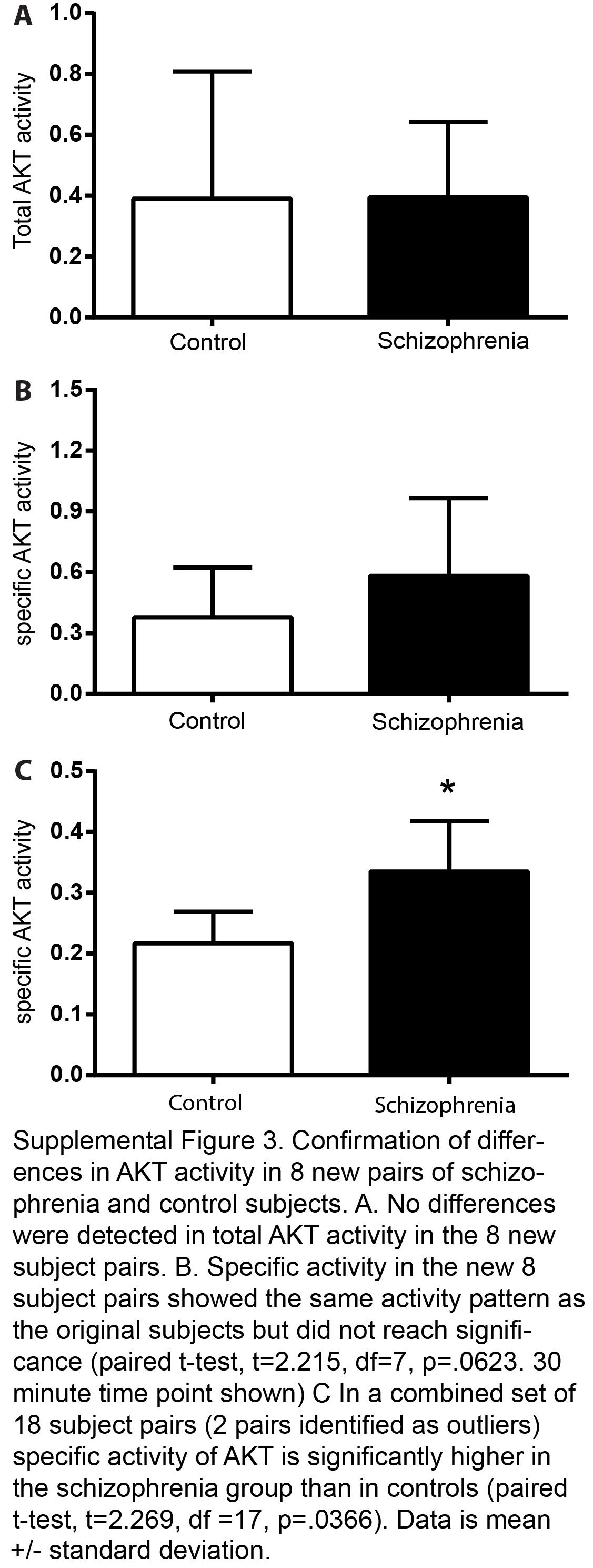
**

**Supplemental methods.**

**Subjects**. Subjects with schizophrenia were diagnosed with this illness for at least 30 years. The brain banking procedures were approved by the Mount Sinai School of Medicine Institutional Review Board. The medical records of the subjects were examined using a formal blinded medical chart review instrument as well as in person interviews with the subjects and/or their caregivers. Schizophrenia subjects were diagnosed based on DSM III-R criteria. Comparison subjects with no history of psychiatric or neurological disease were selected using a formal blinded medical chart review. Exclusion criteria were a history of alcoholism, death by suicide, or coma for more than 6 hours before death. Next of kin consent was obtained for all subjects. The subjects were evaluated for NINCDS-AIREN criteria for a diagnosis of vascular dementia; NINCDS, DSMIV and CERAD diagnosis of dementia; Consensus criteria for a clinical diagnosis of Probable or Possible diffuse Lewy body disease; UPDRS for Parkinson’s disease; clinical criteria for diagnosis of Frontotemporal dementia; medical history of psychiatric disease; history of drug or alcohol abuse; and other tests of cognitive function including the MMSE and CDR. In addition, each brain tissue specimen was examined neuropathologically using systematized macro- and microscopic evaluation using CERAD guidelines. Since the patients in our cohort were elderly at the time of death, many of the subjects have the cognitive impairment associated with aged subjects with schizophrenia ^1-3^*.*

**Kinome array rodent studies**. 130 ug of tissue from the frontal pole of haloperidol-treated and control rats was manually homogenized on ice in Mammalian Protein Extraction buffer with HALT protease and phosphatase inhibitor cocktail (ThermoScientific, Rockford, IL) and stored at -80C until use. Profiling of the serine-threonine subkinome was performed using the PamStation 12 (PamGene International) and STK PamChips ^6^. Briefly, kinase activity was measured in real time using Evolve kinetic image capture software (PamGene) capturing FITC labeled anti-phospho antibodies binding to each phosphorylated substrate every 6 seconds for 90 minutes ^7^. The slope of the exposure brightness over multiple exposure times (multiplied by 100) within the 99th percentile were used to calculate minimal positive shift and data were log_2_ transformed. The signal for each peptide is the log transformed spot intensity. Log transformation allows comparisons of increased and decreased kinase activity on the same scale. Signal intensities were analyzed using BioNavigator 5.2 Software (PamGene).

**Supplemental References**

1 Powchik, P. *et al.* Postmortem studies in schizophrenia. *Schizophr Bull* **24**, 325-341 (1998).

2 Purohit, D. P. *et al.* Severe cognitive impairment in elderly schizophrenic patients: a clinicopathological study. *Biol Psychiatry* **33**, 255-260 (1993).

3 Beeri, M. S. *et al.* Coronary artery disease is associated with Alzheimer disease neuropathology in APOE4 carriers. *Neurology* **66**, 1399-1404, doi:10.1212/01.wnl.0000210447.19748.0b (2006).

4 McCullumsmith, R. E. *et al.* Decreased NR1, NR2A, and SAP102 transcript expression in the hippocampus in bipolar disorder. *Brain Res* **1127**, 108-118, doi:10.1016/j.brainres.2006.09.011 (2007).

5 O'Donovan, S. M. *et al.* Glutamate transporter splice variant expression in an enriched pyramidal cell population in schizophrenia. *Translational psychiatry* **5**, e579, doi:10.1038/tp.2015.74 (2015).

6 McGuire, J. L. *et al.* Altered serine/threonine kinase activity in schizophrenia. *Brain Res* **1568**, 42-54, doi:10.1016/j.brainres.2014.04.029 (2014).

7 Jarboe, J. S. *et al.* Kinomic profiling approach identifies Trk as a novel radiation modulator. *Radiotherapy and oncology : journal of the European Society for Therapeutic Radiology and Oncology* **103**, 380-387, doi:10.1016/j.radonc.2012.03.014 (2012).
